# Inflammation and delayed oral calcium supplementation: Associations with milk yield, reproduction, and herd removal in multiparous Holstein dairy cows

**DOI:** 10.3168/jdsc.2026-1056

**Published:** 2026-06-17

**Authors:** Kathryn D. Bach, Jackson A. Seminara, Julia Poggi, Jessica A.A. McArt

**Affiliations:** 1 Department of Population Medicine and Diagnostic Sciences, College of Veterinary Medicine, Cornell University, Ithaca, NY 14853; 2 College of Agriculture and Life Sciences, Cornell University, Ithaca, NY 14850

## Abstract

The transition from late gestation to early lactation is characterized by metabolic and immune challenges that predispose dairy cows to conditions such as dyscalcemia and excessive inflammation. Both conditions have been associated with impaired health and productivity, and recent evidence suggests they are physiologically linked. Oral calcium supplementation has been proposed as a strategy to support calcium homeorhesis, but results have been inconsistent, possibly due to cow-level factors such as inflammation. The objective of our observational study was to determine whether inflammatory status at 2 DIM, assessed by serum haptoglobin concentrations, modifies the response to oral calcium supplementation administered at 2 and 3 DIM in multiparous Holstein cows. Serum samples and data from 535 cows enrolled in a previously conducted randomized trial on 4 commercial farms in New York were analyzed. Outcomes included average daily milk yield through 15 wk of lactation, pregnancy to first service, time to pregnancy, and time to herd removal. Linear mixed-effects, quasi-Poisson, and Cox proportional hazards models were used to evaluate associations, with marginal means, risk ratios, and hazard ratios calculated at low (0.4 g/L) and high (1.1 g/L) haptoglobin concentrations. For cows with high haptoglobin concentrations at 2 DIM, oral calcium supplementation increased milk yield by 3.1 kg/d and the risk of pregnancy to first service (risk ratio = 1.6, 95% CI = 0.9 to 2.6) in parity ≥3 cows, and improved the hazard of pregnancy (hazard ratio = 1.3; 95% CI: 1.0 to 1.7) and reduced the hazard of herd removal (hazard ratio = 0.5; 95% CI: 0.3 to 1.0) for cows of all parities. No benefit of oral calcium supplementation was observed in cows with low haptoglobin concentrations at 2 DIM other than a 60% increase in risk of pregnancy to first service for parity 2 cows. Our findings suggest that inflammatory status may modify the response to oral calcium supplementation and highlight the potential for targeted strategies to improve postpartum performance.

## Highlights

• Delayed oral calcium supplementation benefited cows with high postpartum inflammation.
• Increased milk production varied by parity; improved reproduction and longevity were consistent.
• Inflammation status might guide targeted calcium supplementation strategies.

## Graphical Abstract

**Figure:**
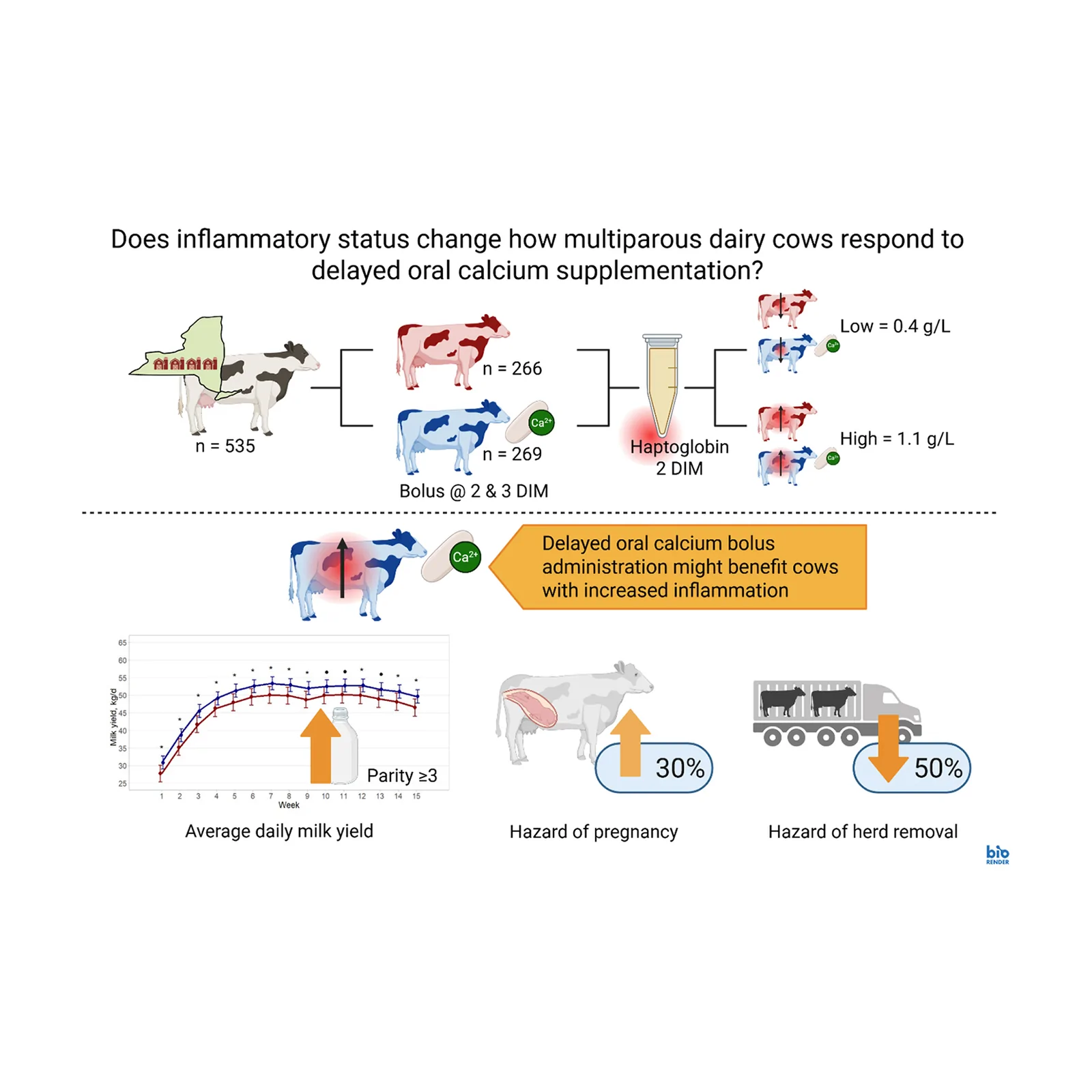
**Summary:** The transition from late gestation to early lactation is characterized by metabolic and immune challenges predisposing dairy cows to conditions such as dyscalcemia and excessive inflammation. Oral calcium supplementation has been proposed to support calcium homeorhesis, but results have been inconsistent. The objective of our observational study was to determine whether inflammatory status at 2 days in milk (DIM) modifies the response to delayed oral calcium supplementation administered at 2 and 3 DIM. For cows with high haptoglobin concentrations at 2 DIM, delayed oral calcium supplementation increased milk yield and improved reproductive and longevity outcomes.

The transition from late gestation to early lactation is characterized by major metabolic and immune challenges in dairy cows. Though many cows navigate this period with success, a subset experience dysregulation of calcium homeorhesis, known as dyscalcemia, and are known to suffer negative downstream health and production outcomes (Couto Serrenho et al., 2021; McArt and Oetzel, 2023; Seminara et al., 2026a). Similarly, cows that experience excessive postpartum inflammation also exhibit increased risk of negative consequences (Huzzey et al., 2009; Nightingale et al., 2015; Seminara and McArt, 2026b). Recent evidence suggests these similarities are not only a coincidence, but that dyscalcemia and excessive inflammation are physiologically associated in these postpartum dairy cows (Couto Serrenho et al., 2025; Seminara et al., 2025). Despite this, few practical strategies exist to mitigate the negative outcomes associated with these dysregulated physiological states.

Over the years, oral calcium supplementation has been evaluated to determine whether it can support postpartum calcium homeorhesis and improve health and production outcomes. However, results have been inconsistent, showing variable benefits and even negative consequences for certain subpopulations of animals (Valldecabres et al., 2023; Couto Serrenho et al., 2024). Given the potential role inflammation plays in altering calcium dynamics, a better understanding of how inflammatory status modifies the response to oral calcium supplementation is warranted. Although the timing of postpartum changes in the calcium-inflammation relationship is still being explored, research suggests measurable differences in markers of systemic inflammation between cows of differing calcium profiles emerge 24 and 72 h postpartum (Couto Serrenho et al., 2025; Graef et al., 2025; Seminara et al., 2026a). Interestingly, administration of oral calcium at calving and 12 h later does not alter serum haptoglobin or albumin concentrations, nor does it affect the relationship between calcium and systemic inflammation in the week following calving (Couto Serrenho et al., 2024, 2025). Therefore, our objective was to determine whether oral calcium supplementation of multiparous dairy cows at 2 and 3 DIM would differentially affect outcomes of cows of varying inflammatory statuses at 2 DIM, assessed using serum haptoglobin concentrations. We hypothesized that cows with elevated haptoglobin would have increased average daily milk yield through 15 wk of lactation, reduced time to pregnancy, and increased time to herd removal if supplemented with oral calcium compared with less inflamed cows.

Our study was a retrospective analysis of a previously conducted randomized control trial on 4 commercial dairy farms in New York evaluating delayed oral calcium supplementation, a 43-g calcium bolus (Bovikalc, Boehringer Ingelheim, St. Joseph, MO) administered at 2 DIM with a second 43-g calcium bolus administered at 3 DIM, on milk production in multiparous Holstein cows (Seely et al., 2024). For data to be included in our study, previously enrolled cows had to have a serum sample collected at 2 DIM before bolus administration and belong to the control (n = 266) or delayed bolus treatment groups (n = 269). Serum samples were stored at −20°C until analysis for haptoglobin at the Animal Health Laboratory at the University of Guelph (Guelph, Ontario, Canada) using a method based on Makimura and Suzuki (1982) on an automated analyzer (Cobas c501, Roche Diagnostics). Intra- and inter-assay coefficients of variation were 2.0% and 8.2%, respectively.

Farm A milked 3,600 cows and farm B milked 1,700 cows, both with a voluntary waiting period (**VWP**) of 74 d, farm C milked 1,800 cows with a VWP of 55 d, and farm D milked 5,000 cows with a VWP of 47 d. All farms milked 3 times a day. Detailed reproductive management can be found in Seely et al. (2024). Briefly, farms A and B enrolled cows into a Presynch breeding program between 36 to 42 DIM followed by initiation of Ovsynch (including a second dose of PGF_2α_ 24 h after the first dose) a week later. Farms C and D enrolled cows into a Presynch breeding program between 40 to 46 DIM and 33 to 39 DIM, respectively. Following Presynch, cows were bred via heat detection for 14 d before enrollment in Ovsynch if no heat was observed. Daily milk yield, time to pregnancy, and herd removal (died or sold) data were extracted from DairyComp 305 (Valley Agriculture Software, Tulare, CA). Daily milk yield was averaged on a weekly basis over the first 15 wk of lactation. Time to pregnancy was defined as the number of days from the end of the VWP to the day of breeding that resulted in a confirmed pregnancy; cows were censored if removed from the herd <30 d after first breeding or if they did not get pregnant within 100 d past the end of the VWP. Herd removal was calculated using the period from calving to 150 DIM.

All descriptive summaries and analytical statistics were analyzed using R version 4.5.1 in RStudio (Posit Software, PBC, Boston, MA). Descriptive summaries were generated using general functions contained in the base package. Continuous variables were summarized as means and standard deviations, medians and interquartile range, minimum, and maximum. Categorical variables were summarized as counts and percentages.

To evaluate milk yield over 15 wk of lactation, a linear mixed effect model was created using the lme4 package (Bates et al., 2015). Based on the scientific question, and to address specific hypotheses regarding effect modification, model structure was established before analysis. To test whether the association between treatment and milk yield over time was modulated by inflammatory status, the 3-way interaction between treatment status (control or treatment), haptoglobin as a continuous variable (g/L), and time as a categorical variable (week of lactation) was included as the primary effect of interest, with farm as a fixed effect. To answer our secondary question as to whether this effect modification varied by parity group, a 3-way interaction between treatment, haptoglobin concentration, and parity group (parity 2 or ≥3), was included as a secondary effect of interest. We anticipated that effect modification by haptoglobin would be nonlinear, consistent with a biological ceiling to the effect of inflammatory burden, and that this biological ceiling would vary by parity group; therefore, an interaction between treatment, the quadratic haptoglobin term, and parity group was also included in the model. Prior data presented by Seminara and McArt (2026b) demonstrate evidence of parity specific modification of the association between haptoglobin concentrations at 2 DIM and milk yield over time. As a result, we included the haptoglobin × parity group × time interaction terms for both linear and quadratic haptoglobin terms in our model to account for this known effect modification and prevent biased estimation of treatment effects. Similarly, as it was assumed that due to known heterogeneity in management, genetics, and environmental conditions, lactation curves would differ across farms, a farm (A, B, C, or D) by time interaction was also included in our model. The model was fit with hierarchical inclusion of all lower order terms and the random effect of cow. By default, the lme4 package assumes a compound symmetric structure within the random effect covariance matrix (Bates et al., 2015). Model assumptions were assessed through visual inspection of diagnostic plots, residuals plotted over the range of predicted values to evaluate the homoscedasticity assumption, and histograms of residuals to evaluate their normality. All model assumptions were satisfactorily met.

To evaluate the relationships between treatment, haptoglobin, parity, and risk of pregnancy to first service, we fit generalized linear models using a quasi-Poisson distribution. With only one observation per animal, the resulting dataset was substantially smaller for this analysis; therefore, we employed a conservative model-reduction strategy to improve parsimony and estimate stability while retaining all prespecified effects of interest and known confounders. The biologically plausible maximal model included the 3-way interactions between treatment, haptoglobin, and parity group, with both a linear and a quadratic haptoglobin term, all constituent lower order terms, and the fixed effect of farm. Noninformative higher order interaction terms (*P* > 0.1) were removed from the model if possible.

For the time to pregnancy and herd removal outcomes, Cox proportional hazards models were fit using the survival package of R (Therneau, 2024). The biologically plausible maximal model included the 3-way interactions between treatment, haptoglobin, and parity group, with both a linear and a quadratic haptoglobin term, all constituent lower order terms, and the fixed effect of farm. Three-way interactions were included in the maximal model to potentially describe treatment effects that differed between cows of different haptoglobin statuses in different parity groups, whereas the haptoglobin quadratic terms were tested within this association under the assumption that the effect of haptoglobin status would not have a linear relationship with the outcome. The proportional hazards assumption was met for both models.

As there is no evidence-based haptoglobin concentration in early lactation that is associated with high or low inflammatory activation, we fit haptoglobin as a continuous variable to use our full range of data and not impose a threshold structure. After models were fit using haptoglobin as a continuous variable, each model was used to calculate proportionally weighted marginal means, risk ratios (**RR**), and hazard ratios (**HR**) as appropriate at 2 reference concentrations of haptoglobin, selected a priori to represent either low (0.4 g/L) or high (1.1 g/L) postpartum inflammatory activation, using the approach described in Seminara and McArt (2026b). These reference concentrations correspond to mean haptoglobin concentrations among diseased and healthy cows (low and high, respectively) from Huzzey et al. (2009). Retrospective classification was not performed. Instead, estimates are model-based calculations at specified haptoglobin concentrations with all other covariates set to proportionally weighted average values. According to the milk production model structure, marginal means were calculated at each inflammatory concentration for each treatment group stratified by parity group using the emmeans package (Lenth, 2024). Pairwise comparisons were conducted between predicted means at each time point and were not adjusted for multiple comparisons across time points due to the exploratory nature of this analysis. According to model structure for pregnancy to first service, RR and 95% CI were calculated between treatment groups, at each inflammatory threshold, for each parity group. The models for both time to event outcomes resulted in calculation of HR and 95% CI between treatment groups at each inflammatory threshold, accounting for parity.

The final dataset included 535 cows with the distribution by farm, parity, treatment, herd removal, and proportion bred shown in Table 1. Haptoglobin concentrations at 2 DIM were right-skewed, with a mean (SD) of 0.7 g/L (0.6 g/L), a median (interquartile range) of 0.5 g/L (0.3 to 0.8 g/L), and a range of 0.1 and 3.6 g/L. Compared with the reference values, 35% of cows (n = 188) had haptoglobin concentrations ≤0.4 g/L, 41% (n = 222) had haptoglobin concentration >0.4 and <1.1 g/L, and 23% (n = 125) had haptoglobin concentrations ≥1.1 g/L. Model summaries for average daily milk yield, pregnancy to first service, time to pregnancy, and time to herd removal are presented in Table 2.

**Table 1:** Descriptive information for 535 multiparous Holstein cows from 4 commercial dairy farms in New York receiving oral calcium supplementation at 2 and 3 DIM (treatment) or not (control)

Farm	Total (%)		Number (%)											
			Parity group				Treatment group				Bred^1^		Removed^2^	
			2		≥3		Control		Treatment					
A	130	(24)	49	(38)	81	(62)	69	(53)	61	(47)	118	(91)	20	(15)
B	93	(17)	37	(40)	56	(60)	44	(47)	49	(53)	88	(95)	5	(5)
C	62	(12)	31	(50)	31	(50)	33	(53)	29	(47)	52	(84)	13	(21)
D	250	(47)	102	(41)	148	(59)	120	(48)	130	(52)	232	(93)	41	(16)
Total	535		219	(41)	316	(59)	266	(50)	269	(50)	490	(92)	79	(15)

1 Defined as bred at least once within 100 d following the VWP.

2 Defined as removed from the herd within 150 DIM.

**Table 2:** Summary of final *P*-values for models used to assess the associations of average daily milk yield during the first 15 wk of lactation, risk of pregnancy to first service, time to pregnancy within 100 d following the VWP, and time to herd removal within 150 DIM for 535 multiparous Holstein cows from 4 commercial dairy farms in New York receiving delayed oral calcium supplementation at 2 and 3 DIM

Item	Outcome			
	First 15 wk milk yield	Pregnancy to first service	Time to pregnancy	Time to herd removal
	Model type			
	Linear mixed effects	Quasi-Poisson	Cox proportional hazards	Cox proportional hazards
	*P*-value			
Covariate				
Treatment	0.4	0.08	0.3	0.3
Haptoglobin	0.4	0.7	0.6	0.08
Parity group	0.4	0.97	0.2	0.04
Farm	<0.001	0.1	0.005	0.006
Time	<0.001	—	—	—
Haptoglobin^2^	0.99	—	—	0.1
Treatment × haptoglobin	0.2	0.4	0.007	0.08
Treatment × parity group	0.1	0.5	—	—
Treatment × time	0.006	—	—	—
Treatment × haptoglobin^2^	0.2	—	—	0.08
Haptoglobin × parity group	0.2	—	—	—
Haptoglobin × time	0.6	—	—	—
Haptoglobin^2^ × parity group	0.3	—	—	—
Haptoglobin^2^ × time	0.98	—	—	—
Farm × time	<0.001	—	—	—
Treatment × haptoglobin × time	0.01	—	—	—
Treatment × haptoglobin × parity group	0.03	0.07	—	—
Treatment × haptoglobin^2^ × parity group	0.05	—	—	—
Time × haptoglobin × parity group	<0.001	—	—	—
Time × haptoglobin^2^ × parity group	<0.001	—	—	—

Average milk yield across the first 15 wk of lactation for the study population was 48.6 kg (SD = 10.5 kg). Daily milk yield averaged by week over the first 15 wk of lactation is illustrated in Figure 1. According to the interaction structure of the model, results were stratified by parity group and treatment status and calculated separately for each inflammatory threshold. Oral calcium supplementation did not strongly influence cows at the low inflammatory concentration in either parity group. At the high inflammatory concentration, cows in parity 2 who received oral calcium supplementation produced numerically less milk than cows who did not receive the treatment at each of the first 15 wk of lactation. When averaged across all weeks evaluated, results provided supportive evidence that cows at the high inflammatory concentration in parity 2 that received oral calcium supplementation produced 2.5 kg/d less milk than cows that did not receive oral calcium supplementation (treatment: 44.7 kg/d, 95% CI = 42.3 to 47.0 kg/d; control: 47.2 kg/d, 95% CI = 45.1 to 49.3 kg/d; *P* = 0.1). Conversely, cows at the high inflammatory concentration in parity ≥3 who received oral calcium supplementation produced more milk than cows that did not receive oral calcium supplementation at wk 1 to 9 of lactation and again at 12, 14, and 15 wk of lactation. Overall, when averaged across all weeks evaluated, parity ≥3 cows at the HI inflammatory concentration who received oral calcium supplementation produced 3.1 kg/d more milk than cows who did not receive the treatment (treatment: 49.0 kg/d, 95% CI = 47.4 to 50.6 kg/d; control: 45.9 kg/d, 95% CI = 43.8 to 48.0 kg/d; *P* = 0.02).

**Figure 1:**
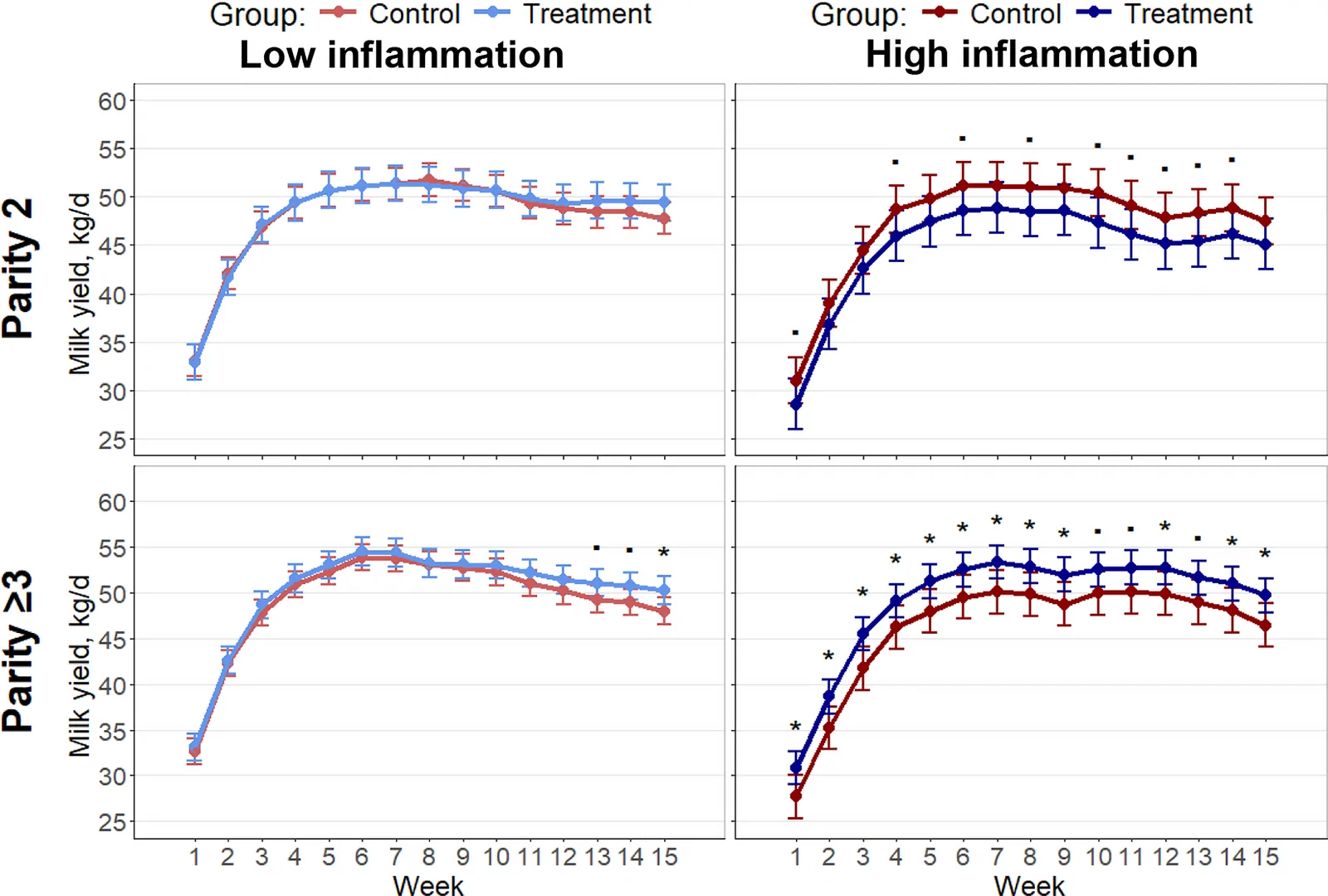
Modeled average daily milk yield during the first 15 wk of lactation for 535 multiparous Holstein cows from 4 commercial dairy farms in New York State receiving oral calcium supplementation at 2 and 3 DIM (treatment: blue points and lines) or not (control: red points and lines) stratified by parity (top row: 2; bottom row: ≥3) and inflammation classification (haptoglobin concentration at 2 DIM; left column: low = 0.4 g/L; right column: high = 1.1 g/L). Dots (•) indicate differences (0.1 ≥ *P* > 0.05) and asterisks (*) indicate differences (≤0.05) between calculated means. Error bars represent 95% CI.

Among all cows that were bred at least once (n = 490), 135 (28%) became pregnant to first service. Of these, 58 belonged to the control group and 77 were cows supplemented with calcium, representing 24% and 31% of each treatment group, respectively. Across parity groups, 27% of parity 2 cows and 28% of parity ≥3 cows became pregnant to first service. With respect to pregnancy to first service, the final model suggested an interaction between haptoglobin concentrations at 2 DIM, parity group, and treatment status (*P* = 0.07); therefore, RR were calculated between treatment groups within each parity group at each inflammatory concentration. In parity 2 cows at the low inflammatory concentration, those that received calcium supplementation were 60% more likely to get pregnant to first service than control cows (RR = 1.6; 95% CI = 1.0 to 2.8; *P* = 0.07), whereas at the high inflammatory threshold, the risk of pregnancy to first service did not differ between groups (RR = 1.3; 95% CI = 0.7 to 2.2; *P* = 0.4). In parity ≥3 cows at the low inflammatory concentration, the RR between treated and control cows was 1.0 (95% CI = 0.6 to 1.5; *P* = 0.99), whereas at the high inflammatory concentration this RR was 1.6 (95% CI = 0.9 to 2.6; *P* = 0.09). Though this evidence is modest from an inferential perspective, a 60% improvement in pregnancy to first service is not marginal, and this evidence is consistent with the premise that response to treatment depends on both inflammatory status at 2 DIM and parity group.

Of the 490 cows bred at least once, 352 (72%) became pregnant within 100 d of the VWP, with a median time to pregnancy for cows that became pregnant of 38 d (interquartile range = 9 to 74 d). By treatment group, 169 of the control cows (69%) and 183 of the treated cows (74%) were pregnant within this timeframe. Across parity groups, 74% of parity 2 cows and 70% of parity ≥3 cows were pregnant within 100 d of the VWP. The treatment by haptoglobin interaction in the time to pregnancy model showed that the effect of treatment depended on haptoglobin status (*P* = 0.007). Results are thus presented at each inflammatory concentration for the whole population controlling for parity group. No difference was observed in the hazard of pregnancy between cows at the low inflammatory concentration that received or did not receive oral calcium supplementation (HR = 1.0, 95% CI = 0.8 to 1.2; *P* = 0.7). At the high inflammatory concentration, however, cows that received oral calcium administration had a greater hazard of pregnancy than nontreated cows (HR = 1.3, 95% CI = 1.0 to 1.7; *P* = 0.02). This finding indicates that cows at the high inflammatory threshold who receive oral calcium supplementation, regardless of parity, became pregnant at a 30% greater rate within 100 d following the end of the VWP than cows that did not receive the treatment.

Fifteen percent of cows were removed from their herd within 150 DIM, with a median time to herd removal for cows that left the herd of 81 d (interquartile range = 52 to 115 d). For the time to herd removal within 150 DIM outcome, the treatment by haptoglobin interaction and the interaction of treatment with the haptoglobin quadratic term provided suggestive evidence that the effect of treatment was influenced by haptoglobin status (*P* = 0.08 for both terms). For cows at the low inflammatory concentration, no difference was detectable between cows that received oral calcium supplementation and control cows (HR = 0.9, 95% CI = 0.5 to 1.5; *P* = 0.7). Cows at the high inflammatory concentration, however, experienced a 50% reduced rate of herd removal within 150 DIM (HR = 0.5, 95% CI = 0.2 to 1.0; *P* = 0.05).

Our results show that oral calcium supplementation at 2 and 3 DIM might benefit cows with increased inflammation. In the case of average daily milk yield, this effect does appear to be modulated by parity, with cows in parity ≥3 showing increased benefit of calcium supplementation compared with parity 2 cows. Similarly, parity ≥3 cows with increased inflammation were more likely to become pregnant to first service following calcium administration, which did not benefit older cows with low inflammation at 2 DIM. Interestingly, parity 2 cows with low inflammation showed the opposite effect, with an increased risk of pregnancy to first service following calcium supplementation which did not benefit parity 2 cows with increased inflammation. This lack of statistical association for parity 2 cows with increased inflammation might be a type II error as power to detect a difference, if one truly existed, was low. Regardless, oral calcium supplementation at 2 DIM did not reduce the risk of pregnancy to first service for any parity or inflammatory group and could be considered as a management strategy given its beneficial effect of a 60% increase in risk of pregnancy to first service for some groups. The benefits of calcium supplementation on longer-term reproduction and longevity, however, appear to be more universal, with an increased hazard of pregnancy and decreased hazard of herd removal for supplemented cows of all parities with high inflammation. Together, our results suggest that inflammatory status modifies the response to delayed oral calcium supplementation.

This concept aligns with recent evidence from Seminara and McArt (2026b) that demonstrates that excessive postpartum inflammation at 2 DIM is associated with reduced lactational success, including reductions in milk production and an increase in herd removal. Similarly, Couto Serrenho et al. (2025) showed a clear association between systemic inflammation and reductions in serum total calcium concentrations during early lactation, a relationship further supported by experimental models where immune activation directly reduced circulating calcium (Al-Qaisi et al., 2020). These findings reinforce the physiological link between inflammation and calcium homeorhesis, suggesting that cows experiencing heightened inflammatory activation may represent a vulnerable subgroup where calcium supplementation might be particularly impactful.

Limitations of our study include the retrospective analysis of data from a trial not originally designed for this question, as well as external validity beyond farms of similar management and geography. It is also important to note that this study assessed calcium supplementation at 2 and 3 DIM, and similar results might not be observed in cows supplemented with calcium at calving or 1 DIM. Additionally, the low number of cows removed from the herd (n = 79) might affect time to event model robustness, and further data are needed to support this finding.

Given the increasing evidence supporting the connection between inflammation, calcium dynamics, and health and performance outcomes, further investigation regarding the mechanisms underlying this relationship within different cow subpopulations might yield a greater understanding of our findings. In addition, exploration of how inflammatory status can inform treatment decisions on farms might provide opportunities for impactful intervention, including investigation into actionable haptoglobin concentration thresholds for decision-making.

In conclusion, our study provides supportive evidence that subpopulations of cows, particularly those with high inflammation and increased parity, might benefit from oral calcium supplementation at 2 and 3 DIM, reinforcing the need for a targeted approach for early lactation management.
